# Celiac Disease: Diagnostic Standards and Dilemmas

**DOI:** 10.3390/diseases3020086

**Published:** 2015-06-16

**Authors:** Dharmesh H. Kaswala, Gopal Veeraraghavan, Ciaran P. Kelly, Daniel A. Leffler

**Affiliations:** The Celiac Center, Division of Gastroenterology, Beth Israel Deaconess Medical Center, 330 Brookline Avenue, Boston, MA 02215, USA; E-Mails: dkaswala@bidmc.harvard.edu (D.H.K.); gveerara@bidmc.harvard.edu (G.V.); ckelly2@bidmc.harvard.edu (C.P.K.)

**Keywords:** celiac disease, diagnosis, serology

## Abstract

Celiac Disease (CD) affects at least 1% of the population and evidence suggests that prevalence is increasing. The diagnosis of CD depends on providers being alert to both typical and atypical presentations and those situations in which patients are at high risk for the disease. Because of variable presentation, physicians need to have a low threshold for celiac testing. Robust knowledge of the pathogenesis of this autoimmune disease has served as a catalyst for the development of novel diagnostic tools. Highly sensitive and specific serological assays including Endomysial Antibody (EMA), tissue transglutaminase (tTG), and Deamidated Gliadin Peptide (DGP) have greatly simplified testing for CD and serve as the foundation for celiac diagnosis. In addition, genetic testing for HLA DQ2 and DQ8 has become more widely available and there has been refinement of the gluten challenge for use in diagnostic algorithms. While diagnosis is usually straightforward, in special conditions including IgA deficiency, very young children, discrepant histology and serology, and adoption of a gluten free diet prior to testing, CD can be difficult to diagnose. In this review, we provide an overview of the history and current state of celiac disease diagnosis and provide guidance for evaluation of CD in difficult diagnostic circumstances.

## 1. Introduction

Celiac disease (CD) is a chronic immune-mediated enteropathy triggered by exposure to gluten in genetically predisposed individual [1] and is a common autoimmune disorder, affecting ~1% of the population in many regions of the world [2,3]. CD is genetically based and prevalence is enriched in patients with family history of CD or a personal history of autoimmune disease, including thyroid, liver, and type 1 diabetes mellitus [4]. Symptoms of undiagnosed CD can range from subclinical to severe malabsorption, known as celiac crisis [5].

## 2. Who Should be Tested for Celiac Disease

**Figure 1:**
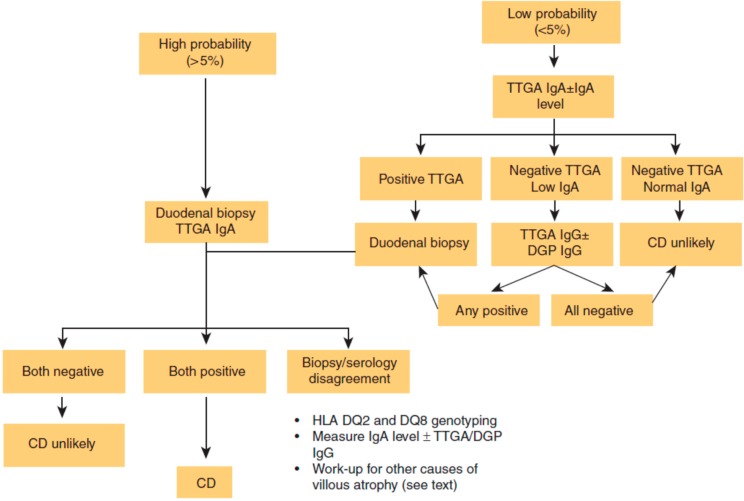
Celiac disease (CD) diagnostic algorithm. DGP: deamidated gliadin peptide; HLA: human leukocyte antigen; Ig: immunoglobulin; TTGA: tissue transglutaminase antibody [7]. Reproduced with permission from Kelly C.P. [7], (ACG clinical guidelines: Diagnosis and management of celiac disease-3).

The most important reason for the relatively low rate of CD diagnosis [3] is failure to consider testing. CD is often not considered due to its wide range of clinical presentation. CD diagnosis rate is increasing due to both increased true prevalence [6] and improved awareness of its variable clinical presentation [7]. Through the 1950s, diagnosis was based on malabsorptive features and clinical observation. Development of intestinal biopsy, initially by Crosby capsule and later by endoscopy, subsequently became the gold standard for confirmation of CD diagnosis, a position it has kept to this day [8]. Serologic tests are generally recommended as the first step when there is suspicion of CD in order to identify patients who should undergo intestinal biopsy. Recommendations are now available to help clinicians decide which patients should be tested for CD (Table 1) [9] and how to proceed with evaluation of potential CD. (Figure 1) [7].

**Table 1:** Who should be tested for CD [9].

High Risk Patients Routinely Test for CD: Consider Endoscopy even if Serology Negative	Medium Risk Patients Consider CD Serologic Testing: CD Sufficiently Excluded if Serology Negative	Low Risk Patients Consider testing if refractory to standard therapy or other clinically unusual features: CD sufficiently excluded if serology negative
(1) Chronic gastrointestinal symptoms with a family history of celiac disease or a personal history of autoimmune disease or IgA deficiency (2) Biopsy proven dermatitis herpetiformis (3) Chronic diarrhea (4) Failure to thrive in children (5) Iron deficiency anemia refractory to oral supplementation	(1) Irritable bowel syndrome (2) Elevated liver function tests (3) Iron deficiency anemia (4) Fatigue/lethargy (5) Chronic gastrointestinal symptoms without a family history of celiac disease or a personal history of autoimmune disease (6) Peripheral neuropathy (7) Ataxia (8) Dental enamel defects (9) Recurrent aphthous ulcerations (10) Hyposplenism (11) Fertility abnormalities (12) Down’s or Turner’s syndrome (13) Known IgA deficiency (14) Microscopic colitis	(1) Osteopenia/osteoporosis (2) Fibromyalgia (3) Chronic Fatigue Syndrome (4) Heartburn/GERD (5) Acute or chronic pancreatitis (6) Alopecia (7) Myalgias/Arthralgias (8) Autoimmune liver disease (9) Personal history of autoimmune disease or connective tissue disease without ongoing unexplained symptoms (10) Skin lesions other than dermatitis herpetiformis (11) Headaches including migraines (12) Mood disorders (13) Attention deficit disorder/cognitive impairment (14) Epilepsy (15) Restless leg syndrome

Reproduced with permission from Leffler D.A. Celiac disease diagnosis and management: A 46-year-old woman with anemia. *JAMA* **2011**, *306*, 1582–1592 [9].

## 3. Current Diagnostic Guidelines for Celiac Disease

Four guidelines on CD diagnosis have been published by gastrointestinal organizations since 2012. All guidelines include the combined use of biopsy and serologic analyses for diagnosis. According to American College of Gastroenterology (ACG) 2013 CD guidelines combination of both small intestinal biopsy and serologic tests (anti-tissue transglutaminase (tTG) or anti-deamidated gliadin peptide (DGP)) are recommended for diagnosis of CD. This guideline also recommends that children age <2 years should have IgG DGP test due to the lower sensitivity of tTG in this population, discussed in detail below [7]. A consensus guideline in 2012 by the European Society of Pediatric Gastroenterology, Hepatology and Nutrition (ESPGHAN) proposed a non-invasive method of diagnosing CD in select pediatric patients. The ESPGHAN algorithm suggested that, in pediatric patients who have symptoms consistent with CD can be diagnosed without biopsy confirmation if they have an IgA tTG titer >10-fold above the upper limit of normal, a positive endomysial antibody (EMA) in a separate blood sample, and carry the HLA DQ2 or DQ8 haplotype [10]. The British Society of Gastroenterology recommendations for adult CD diagnosis suggest that serologic tests, either tTG, EMA, or DGP should be done as the first step in diagnosis, followed by small intestinal biopsy is a definitive test to diagnose CD. This guidelines suggests that duodenal biopsy cannot currently be replaced by lone serologic testing [11]. Recent guidelines for adult CD patients from the World Gastroenterological Association recommend serologic tests including anti-tTG and/or anti-EMA, or anti-DGP for diagnosis and biopsy suggested but not considered mandatory for CD diagnosis which is appropriate for countries with limited healthcare resources [12]. The guidelines overlap substantially with the major difference between all four guidelines being that ACG and BSG mandates intestinal biopsy to confirm the diagnosis of CD, while ESPGHAN and WGO allow diagnosis of CD without biopsy in certain conditions.

## 4. Serologic Tests

A new era in CD diagnosis began in 1980s with the identification of IgG and IgA anti-gliadin antibodies (AGAs) circulating in plasma of untreated patients [13]. This was a major step forward as prior to this development, CD could be diagnosed only on the basis of clinical suspicion and intestinal biopsy. Binding of Immunoglobulin A (IgA) and IgG antibodies to native gliadin (AGAs), were found to be associated with CD and were the first serologic tests for CD used to inform the need for duodenal biopsy. However, as antibodies to foreign proteins, at least at low titers, are common in healthy individuals, the accuracy of AGA tests are limited, with sensitivity and specificity generally less than 90% and positive predictive values less than 50% in many populations [14]. AGA testing largely fell out of favor in the 1990s as more accurate testing became widely available [15].

In early 1990s, immunofluroscent assays of IgA binding in active CD serum against monkey esophagus endomysium (EMA) was found to be a highly specific and sensitive marker of CD [14,16,17]. Although EMA has a specificity and sensitivity of >95% [18], EMA has thus been suggested to be less sensitive, especially among celiac children under two years of age [19], as well as in elderly patients [18]. Moreover, it has also been reported that false negative EMA results may be associated with milder small-bowel mucosal lesions [18,20]. Despite the high accuracy of EMA, it has the disadvantages that testing is expensive, subjective, and labor-intensive, requiring experienced personnel to perform [2]. While in a reference lab, EMA remains the most sensitive CD test, the technical disadvantages which result in significant inter-observer and inter-site variability have led to EMA being largely replaced by newer enzyme-linked immunosorbent assay (ELISA) based assays [16,17].

In 1997, research studies identified the ubiquitous enzyme tTG as the auto antigen, which reacts with EMA, leading to the development of ELISAs that detect antibodies against tTG [16]. tTG assays quickly demonstrated high sensitivity and specificity with lower cost and greater reproducibility than immunofloroscence assays. Although performance characteristics of assays vary, overall tTG testing is reliable and inexpensive and for these reasons, has become the most common test for celiac diagnosis and monitoring [16,21,22].

Most recently, testing for antibodies against DGP has become clinically available. AGAs are produced in response to gliadin, a prolamin found in wheat. DGP is based on the conversion of gluten peptides to deamidated peptides by the action of intestinal tTG [23,24]. These peptides bind with high affinity to human leukocyte antigen DQ2 or DQ8 on celiac patients’ antigen-presenting cells which stimulate the inflammatory T-cell response observed in the intestinal mucosa of patients with CD [14]. This results in an antibody response to DGP that displays a higher specificity and specificity for CD than antibodies to native gluten (AGAs) [23], and are especially useful in children younger than two years of age [20]. In patients over two years of age IgA-tTG antibody is the preferred serological diagnostic test for diagnosing CD [25]. On the other hand, IgG anti-tTG testing has disappointing sensitivity [24], and IgG anti-DGP and the composite IgA/IgG anti-DGP reach sensitivities above 80% and, importantly, specificities above 95% [14,16,21]. For this reason, IgG-DGP tests are the most accurate available assays for patients with selective IgA deficiency. According to the study high concentration of DGP antibody found to be correlated with severity of intestinal damage in infants and may also help assess dietary adherence [26,27].

Most recently point of care (POC) finger-stick [28,29] blood tests have been introduced for rapid detection of CD. POC tests based on transglutaminase 2 (TG2) auto-antibodies are now available and may be of particular use when laboratory based testing is not readily available [13,29,30]. The newest generation of POC test is based on a combination of DGP and tTG together and has a reported sensitivity of 93.1% and specificity of 95% [31,32]. A combination of immunoassays is particularly useful as an addition to detection of patients with CD who are IgA deficient, however IgG-DGP was able to detect a small number of IgA-sufficient patients who are seronegative for IgA-tTG [20].

Serologic tests in CD should be done as first step in patients with symptoms suggestive of CD and those with CD-associated diseases. However, when clinical suspicion for CD is high, small intestinal biopsies should be considered even in patients with negative serology results.

Intestinal fatty acid binding protein (I-FABP) is a cytosolic protein, which is released by necrotic enterocytes, first described as a marker of intestinal ischemia. I-FABP has been studied as possible marker to evaluate mucosal damage and it is proposed as a sensitive diagnostic test in the evaluation of ischemia in mechanical small bowel obstruction [33,34]. The expression of I-FABP is primarily limited to epithelial cells of the ileum of small intestine. I-FABP is abundantly present in enterocytes and has been reported to be a sensitive marker for damage to the intestinal epithelium [33]. In children with a positive serological test for CD I-FABP has demonstrated positive predictive value of 98% [35]. If validated and clinically available, serum I-FABP might be useful in clinical practice for identifying noncompliance and unintentionally gluten intake, evaluating new therapeutic options, as a non-invasive marker for detection of ongoing mucosal architectural abnormalities without the need of endoscopy and as an aid for celiac diagnosis during gluten challenge [33,34].

## 5. Genetic Testing

Nearly all individuals with CD carry the type II class Human Leukocyte Antigen (HLA) DQ2 and/or DQ8 haplotypes. These molecules are requisite for the high affinity binding of deamidated gluten peptides necessary to generate an immune response [36,37]. Less than 1% of the CD population carries half of the HLA-DQ heterodimer [36]. However, HLA DQ2 and DQ8 are highly prevalent, and can be found in 20%–40% of the general population [37,38]. For this reason, it is estimated that only 3% of individuals with these haplotypes will go on to develop CD giving this test a very low positive predictive value [39,40]. At the same time, HLA typing has a negative predictive value of >99% and is useful for ruling out CD in patients on a gluten free diet (GFD), for patients with an uncertain diagnosis of CD as recommended by ESPGHAN [10], and for risk stratifying CD in at risk family members. Although only one third of family members will be spared repeated testing, particular combinations (e.g., homozygocity for DQ2) increase risk for CD to up to 40% [38,41,42]. Overall however, HLA testing has a positive predictive value generally under 50% depending on the population tested [36].

## 6. Endoscopy and Histology

The diagnosis of CD is based on patient’s symptoms, CD-specific antibody levels, the presence of HLA-DQ2 and/or HLA-DQ8, and characteristic histological changes (villous atrophy and crypt hyperplasia) in the duodenal biopsy. Despite the existence of highly sensitive serological assays, small-bowel mucosal biopsy is still considered the definitive method for diagnosis of CD [43].

Classical CD histologic findings include crypt hyperplasia, blunted or atrophic villi, and increased number of intra-epithelial lymphocytes (IELs), especially at the villous tips (Table 2 Marsh Classification). In addition to these findings, additional histopathological changes are common in CD including neutrophilic and eosinophilic infiltrates, subepithelial collagen thickening and associated lymphocytic gastritis [43,44,45].

**Table 2:** Comparison of Marsh modified (Oberhuber) Histological classification and Villanacci classification of Celiac Disease.

Histologic Findings	Marsh 0	Marsh I	Marsh II	Marsh IIIa	Marsh IIIb	Marsh IIIc
IEL/100 Enterocytes(EC)	<40/100EC	>40/100EC	>40/100EC	>40/100EC	>40/100EC	>40/100EC
Villous atrophy	None	None	None	PVA	STVA	TVA
Crypt Hyperplasia	None	None	Hyperplastic	Hyperplastic	Hyperplastic	Hyperplastic
Villanacci Classification	Type 0	Type A	Type B			

While histologic changes may be obvious, the location, number, and size and orientation of biopsies can affect diagnostic yield. As 70% of cases have patchy mucosal damage [46], it is important to maximize diagnostic accuracy by collecting ≥5 duodenal biopsies with two samples from the duodenal bulb. According to recent studies as many as 13% of patients demonstrated characteristic enteropathy only located to duodenal bulb [46,47]. Endoscopic staining using dye like indigo carmine or methylene blue and water immersion as been suggested to allow visualization of villi and identification of patchy areas however the benefit of this beyond the standard five or more biopsies is unclear [48,49]. Biopsies from the duodenal bulb should be carefully interpreted, because peptic injury can damage villi and be mistaken for CD in some cases [44]. While CD does account for at least 90% of enteropathy in western countries [6], histological features of CD are not specific and are also associated with disorders like giardia infection, common variable immune deficiency, Crohn’s disease, and *Helicobacter pylori* infection [50]. This issue is discussed further below in the section on diagnostic dilemmas.

A simplified histologic classification is also proposed by Villanacci *et al.* [51], based on villous morphology and IEL count. Type A represents the non-atrophic type which is defined as villous crypt ration 3:1, >25 IELs × 100 epithelial cells. Type B is the Atrophic type which is defined as villous crypt ration <3:1, >25 IELs × 100 epithelial cells.

## 7. Endoscopic Markers of Celiac Disease

**Figure 2:**
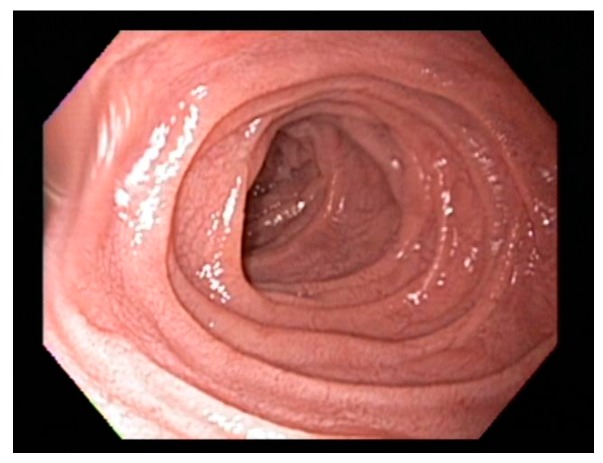
Classical scalloping of duodenal mucosa seen in Celiac disease at endoscopy.

Scalloping folds, mosaic pattern and decrease of duodenal folds are typical endoscopic markers of villous atrophy however should not be relied upon in practice. Studies suggest that the overall specificity and sensitivity of gross endoscopic findings ranges from 83% to 100%, and from 6% to 94%, respectively [50,51] and normal appearance of the duodenum should not preclude biopsy [51,52]. On the other hand, the scalloped feature (Figure 2) of duodenal folds has a positive predictive value of 69% for CD and 96% for enteropathy and should always prompt biopsy when endoscopy is performed for other indications [53].

Video Capsule Endoscopy (VCE) has also been evaluated for use in CD diagnosis. VCE is a good alternative in patients who refuse upper endoscopy, for cases with negative duodenal biopsy and positive serology to assess for distal enteropathy, and to evaluate patients with non-responsive disease, to investigate for complications, such as ulcerative jejunitis or neoplasia. The lack of ability to biopsy is the main limitation of VCE [54].

## 8. Dilemmas in Diagnosis of Celiac Disease

In general, CD diagnosis can be readily established with currently available tests and diagnostic procedures. However, in some patients, the diagnosis is not straightforward and presents a challenge to clinicians. Potential dilemmas include those with positive serology but normal histology, negative serology but abnormal duodenal mucosal histology, and adoption of the GFD prior to testing. In the following section we discuss common dilemmas in diagnosis of CD.

### 8.1. Positive Serology with Normal Biopsy

Positive IgA tTG serology may be seen in in patients with normal small intestinal histology. False positive tTG results are rare but do occur and are usually low titer (less than twice the upper limit of normal). Hypergammaglobuliniemia, chronic liver disease, congestive heart failure and enteric infections have been associated with false positive results [14]. This was a more frequent issue with early generations of tTG assays, which were more liable to cross-react with other antigens.

We suggest that the initial step in evaluation of an individual with elevated tTG titers and normal histology should be review of the biopsies by a gastrointestinal pathologist familiar with CD to look for subtle abnormalities and to confirm that the biopsies are of sufficient number and orientation. If the histology is convincingly normal, we confirm that the patient was on a full gluten containing diet at the time of endoscopy. If the patient was on a low gluten diet, we recommend gluten challenge, discussed below. If these steps do not reconcile results HLA typing should be considered, though, in our experience, patients with high titer IgA-tTG are nearly always positive for HLA DQ2 or DQ8. We also check DGP and/or EMA antibodies in these cases. If more than one celiac serologic test is positive, it strengthens the argument that the patient has a true, if mild, form of CD. After evaluation many of these patients will be found to have potential CD. Potential CD is defined as positive serologic titers with a normal biopsy [1]. It is controversial whether these patients benefit from the gluten free diet. Limited data suggest that patients with mild villous atrophy appear to benefit from a gluten-free diet, but many of these patients are minimally symptomatic [54,55]. We typically recommend a six-month trial of a GFD in this scenario and if there is any clinical benefit, or if there are other clinical signs of CD, such as osteopenia or nutritional deficiencies, the GFD should be continued. If there is no clinical benefit from the GFD, patients may be reasonably followed on a regular diet and reevaluated if tTG titer increases or symptoms change. It has been recently shown that CD markers disappear in many symptomless children at family risk of CD with potential CD left on an normal diet [56]. Spontaneous normalization of tTG has been also demonstrated in children with type 1 diabetes mellitus [57]. Therefore, in symptomless children with positive celiac-type serology, the decision to perform the biopsy may be preceded by repeat serological testing at three to six months when the antibody titer is not very high.

### 8.2. Normal Villous Architecture with Duodenal Lymphocytosis

Although increased intraepithelial lymphocytes (IELs), defined as >25 IELs/100 enterocytes, is recognized as potentially consistent with CD, by itself, this finding lacks specificity. This histological finding can be found in CD, but more commonly with other disorders and medications that cause small intestinal inflammation. Reported etiologies of lymphocytic infiltration of the intestinal epithelium in the absence of villous atrophy include non-steroidal anti-inflammatories, proton pump inhibitors, small intestinal bacterial over growth, *helicobacter pylori* infection, inflammatory bowel disease, and eosinophilic gastroenteritis [58]. About 2.5% of proximal small intestinal mucosal biopsies [59] display increased IELs in the absence of villous architectural change [60]. Determining the etiology of increased intraepithelial lymphocytosis can be challenging and relies on assessment of clinical, serological, and histopathological data [56,57,61].

In general, patients with this finding should have celiac serology tested but if these are negative, CD can be confidently ruled out in most cases.

### 8.3. Negative Serology with Duodenal Biopsy Consistent with Celiac Disease

While modern celiac serologies are highly sensitive, a small percentage of patients with CD will be seronegative at diagnosis [14] and others may show an extremely slow resolution of histological findings, despite a gluten free diet [62], making the diagnosis uncertain. Most patients with villous atrophy on duodenal biopsy will have a serologic test consistent with CD. Differentiation of seronegative CD from alternate causes of enteropathy is a clinical challenge and requires integration of clinical, genetic, and histopathologic criteria [63]. The major possible etiologies in patients with villous atrophy but negative celiac serologies, include patients on a GFD at the time of testing, IgA deficiency, seronegative CD and non-celiac enteropathy(NCE). Celiac-like histological features can be seen with multiple other gastrointestinal disorders including peptic duodenitis [64], Common Variable Immune deficiency (CVID) with malabsorption or anemia [65], and food allergies or eosinophilic gastroenteritis [43,49,62]. Other etiologies include post-viral enteropathy, autoimmune enteropathy, tropical sprue, NSAID enteropathy, and immune mediated enteropathy, all of which may present with negative celiac serology and histology consistent with CD [50].

The histopathological changes of small intestinal Crohn’s disease are heterogeneous and involve varying degrees of acute and chronic inflammation similar to CD [66]. Inflammatory bowel disease has been reported as the second most common cause of villous atrophy after CD [67]. In patients where the diagnosis is uncertain or findings are atypical, intestinal biopsies should be reviewed by a skilled gastrointestinal pathologist. If the initial biopsies are unavailable or prove to be non diagnostic after re-evaluation, repeat endoscopy with biopsy should be performed.

In patients with villous atrophy and negative celiac serology (tTG-IgA) our typical next step is to review the pathology, obtain HLA DQ2/DQ8 and DGP testing and consider causes of NCE [36,50]. If this is non-diagnostic for CD we will check serum immunoglobulins. If serum immunoglobulin reveals low IgG and IgA in setting of normal albumin this suggests CVID [65]. As a part of serologic assessment, IgG-based DGP testing should be considered in all patients with Ig A deficiency because they have a 10–20 times greater risk of developing CD [25,64].

### 8.4. Patients on a GFD Prior to Testing Challenge

One of the most common and vexing clinical issues in celiac diagnosis is the evaluation of patients who adopt the GFD prior to formal testing. In this scenario, the best approach is to start the diagnostic process with serologic tests (anti-tTG and/or DGP) and HLA typing. Seropositivity indicates that the patient is eating sufficient gluten to have active CD and duodenal biopsy is the next step in these cases. However if the serologic tests are negative, this should be followed by genetic testing for HLA DQ2/DQ8, which, if negative, excludes current or future CD.

**Figure 3:**
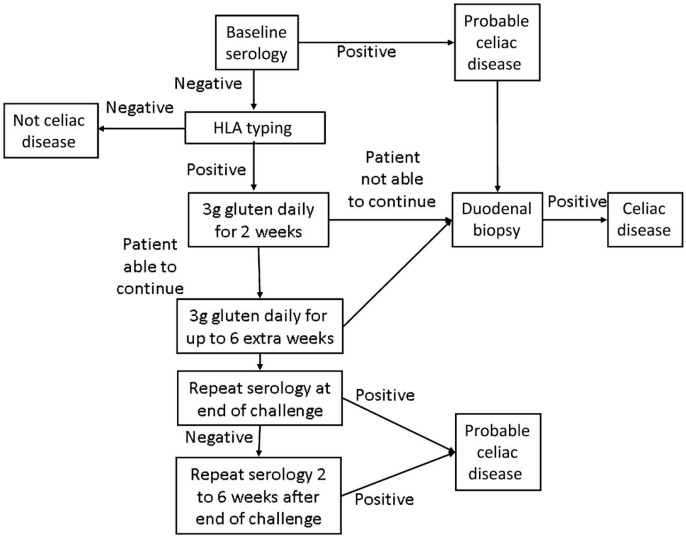
Modified Gluten Challenge Algorithm [68]; Reproduced with permission from Leffler D.A. and Kelly C.P. (Kinetics of the histological, serological and symptomatic responses to gluten challenge in adults with coeliac disease. *Gut* **2012**).

Only once the patient is found to be seronegative and HLA DQ2 and/or DQ8 positive, should gluten challenge for diagnosis be undertaken. (Figure 3) [36,42]. In the past, gluten challenge guidelines suggested intake of at least 10 g of gluten per day for a period of eight weeks or longer. Recent studies have helped to refine the timing, doses, and duration of the gluten challenge [38,66,67]. According to these studies two weeks of gluten challenge can result in mucosal damage. Additionally, a lower level of gliadin (2–3 g/day, equivalent to one to two slices of bread) appears better tolerated and sufficient for diagnosis. (Figure 3) [68]. While this reduced dose and duration gluten challenge significantly reduces the burden of testing, it is still difficult for many patients to complete, and may carry some risk in children and patients with celiac-related neurological manifestations. Further, extended exposure of six months or more may be necessary in a minority of patients. For these reasons, work is being done on alternate methods of diagnosis including *in vitro* gluten challenge [69] and HLA tetramer testing [36] , however none are clinically available at this time.

### 8.5. Diagnosis in Infants and Young Children

CD can present at any age where there is exposure to gluten, including infancy. In early childhood, classic gastrointestinal symptoms of CD are most common, but other presentations including asymptomatic growth delay are also recognized [10,68]. As the immune system is not fully mature, serologic tests are not as sensitive in young children as they are in older children and adults. For many years, AGA testing was considered to be the best test for children under the age of two years [27]. However, in this population DGP antibodies have been found to be more accurate than both AGA and tTG testing [7,42,70]. In young children suspected of having CD, it is reasonable to check both tTG and DGP. If titers are very high and the child otherwise meets ESPGHAN criteria described above, the diagnosis of CD can be confidently made. However, if there are any atypical manifestations, if serologic tests are normal but the child is considered at high risk, or if serologic titers are positive but not highly elevated, endoscopy should be considered.

## 9. Conclusions

CD is a chronic systemic immune-mediated disorder associated with variable small-intestinal mucosal injury triggered by gluten ingestion in genetically predisposed individuals. It is a common disorder, affecting individuals worldwide, with recent studies suggesting an increase in prevalence. Small-bowel biopsy currently remains the gold standard for diagnosis of CD. The improved sensitivity and specificity of serologic testing and growing awareness among physicians should increase the diagnosis of CD. Due to the changing presentation of disease, as well as the recognition of a number of potential histopathologic mimics, communication between pathologists and gastroenterologists is essential for appropriate interpretation of small-bowel biopsy specimens. The clinical, histologic, and laboratory data need to be assessed to provide an explanation for atypical manifestations of CD and possible differential diagnoses. While CD remains a morbid disease, awareness of disease risk factors, current diagnostic testing recommendations and a framework for the evaluation of common diagnostic issues can result in earlier and more accurate diagnosis.
